# Functional MRI activation in white matter during the Symbol Digit Modalities Test

**DOI:** 10.3389/fnhum.2014.00589

**Published:** 2014-08-04

**Authors:** Jodie R. Gawryluk, Erin L. Mazerolle, Steven D. Beyea, Ryan C. N. D'Arcy

**Affiliations:** ^1^Department of Psychology/Neuroscience, University of VictoriaVictoria, BC, Canada; ^2^Faculty of Medicine, Department of Radiology, University of CalgaryCalgary, AB, Canada; ^3^Biomedical Translational Imaging Centre, IWK Health CentreHalifax, NS, Canada; ^4^Applied Sciences, Simon Fraser UniversityBurnaby, BC, Canada; ^5^Fraser Health Authority, Surrey Memorial HospitalSurrey, BC, Canada

**Keywords:** neuroimaging, functional magnetic resonance imaging, white matter, Symbol Digit Modalities Test, neuropsychology

## Abstract

**Background**: Recent evidence shows that functional magnetic resonance imaging (fMRI) can detect activation in white matter (WM). Such advances have important implications for understanding WM dysfunction. A key step in linking neuroimaging advances to the evaluation of clinical disorders is to examine whether WM activation can be detected at the individual level during clinical tests associated with WM function. We used an adapted Symbol Digit Modalities Test (SDMT) in a 4T fMRI study of healthy adults.

**Results**: Results from 17 healthy individuals revealed WM activation in 88% of participants (15/17). The activation was in either the corpus callosum (anterior and/or posterior) or internal capsule (left and/or right).

**Conclusions**: The findings link advances in fMRI to an established clinical test of WM function. Future work should focus on evaluating patients with WM dysfunction.

## Introduction

The ability to measure functional magnetic resonance imaging (fMRI) activation in white matter (WM) using blood oxygen level dependent (BOLD) contrast has potential to advance the clinical investigation of WM disorders (e.g., multiple sclerosis, diffuse axonal injury resulting from brain trauma). A key step in this respect is to examine whether neuropsychological tests that are associated with WM dysfunction can in fact elicit WM fMRI activation at the individual level.

To date, imaging methods have documented structural changes in WM and have attempted to link such changes to behavior (Anzola et al., 1990; Sperling et al., 2001; Charil et al., 2003; Ranjeva et al., 2006). Yet, in many cases, structural measures of WM integrity do not correlate with functional deficits experienced by the patient (i.e., the clinico-radiological paradox; e.g., Pelletier et al., 2009). Functional MRI can provide a more direct assessment, however, the concept of WM activation is controversial (Logothetis and Wandell, 2004). The prevailing assumptions that go against WM fMRI activation relate to two main issues: (1) fMRI signal in WM is thought to be near or below detection thresholds because the cerebral blood flow/volume are lower in WM than gray matter (Rostrup et al., 2000; Preibisch and Haase, 2001; Helenius et al., 2003; Wise et al., 2004; Van Der Zande et al., 2005); and (2) fMRI signal is thought to arise primarily from post-synaptic potentials in gray matter rather than action potentials in WM (Logothetis et al., 2001).

Indeed, there are a number of possible factors that may contribute to the detection of fMRI signal in WM. First, ion channels (e.g., Na^+^/K^+^) in unmyelinated axons and the nodes of Ranvier in myelinated axons have metabolic requirements that must be met (Tettamanti et al., 2002). Secondly, astrocytes, which are known to exist in WM (Orthmann-Murphy et al., 2008; Sun et al., 2010), have been proposed to be functionally entrained to metabolic requirements related to neurotransmitter reuptake/recycling and regulating “cerebral blood delivery” (Figley and Stroman, 2011). Specifically, it is possible that astrocytes, which have end feet on arterioles, mediate vasodilation in WM as a result of increased K^+^ uptake associated with spiking activity (Kalsi et al., 2004; Petzold and Murthy, 2011). Thirdly, it is also possible that NO producing neurons, which have been found in WM, yield a hemodynamic response (Barbaresi et al., 2013). Finally in terms of measurement sensitivity, BOLD signal has previously been detected in WM tissue during vascular challenges (e.g., breath-hold or hypercapnia) (Rostrup et al., 2000; Preibisch and Haase, 2001; Helenius et al., 2003; Macey et al., 2003; Van Der Zande et al., 2005; Mandell et al., 2008; Driver et al., 2010; Thomas et al., 2014).

Given the physiological viability of fMRI activation in WM, there are a rising number of studies from our group and others report WM activation (Maldjian et al., 1999; Tettamanti et al., 2002; Omura et al., 2004; Weber et al., 2005; D'Arcy et al., 2006; Zeffiro et al., 2007; Baudewig et al., 2008; Mazerolle et al., 2008, 2010; Gawryluk et al., 2009, 2011; Yarkoni et al., 2009; Newman et al., 2010; Fabri et al., 2011; Weis et al., 2011).

The ability to detect WM fMRI activation has clear implications for the evaluation of WM disease or damage. In order to prepare for clinical applications, research on WM fMRI must fulfill the following two criteria: (1) be linked to well-known clinical tests and (2) be demonstrable at the individual level.

With regards to the first criterion, neuropsychological testing has shown that patients with WM disease (e.g., multiple sclerosis) present with impairments on measures of attention, memory, and executive function (Wishart et al., 2001; Hoffmann et al., 2007; Rogers and Panegyres, 2007; Chiaravalloti and Deluca, 2008; Smith et al., 2011). The most common and profound cognitive deficits associated with WM disorder are evident on measures of information processing speed (Hoffmann et al., 2007; Rogers and Panegyres, 2007; Chiaravalloti and Deluca, 2008; Smith et al., 2011). Accordingly, one of the most common tests for assessing WM disorders is the Symbol Digit Modalities Test (SDMT; Hoffmann et al., 2007). The SDMT is considered a robust diagnostic measure sensitive to cognitive impairment(s) across WM disorders (Felmingham et al., 2004; Hoffmann et al., 2007; Rogers and Panegyres, 2007; Chiaravalloti and Deluca, 2008). The SDMT has been previously modified for use with fMRI (e.g., Genova et al., 2009). In addition to the gray matter activation, at least one study has published figures depicting evidence of WM activation using the SDMT. While not reported in text, Genova et al. (2009) showed greater activation in the anterior corpus callosum and internal capsule for healthy controls relative to multiple sclerosis patients (see **Figure 3**, Genova et al., 2009). In fact, the corpus callosum and internal capsule are both regions that are functionally consistent with the task demands. Although the SDMT is not an interhemispheric transfer task *per se*, it is likely that information is transferred because both hemispheres are involved in the task [36]. Furthermore, previous DTI studies have shown that multiple sclerosis patients with low fractional anisotropy values in the corpus callosum have impaired performance on the SDMT (Yu et al., 2011). Activation in the posterior limb of the internal capsule is also likely to be task related given the involvement of the corticospinal tract in movement and the required motor response on the fMRI-adapted task. Even when present in the data, WM activation is often not reported. Rather, the result is often either ignored or dismissed as an artifact.

In terms of the second criterion, a growing number of studies have demonstrated white matter activation at the group level (e.g., Gawryluk et al., 2009, 2011; Yarkoni et al., 2009; Mazerolle et al., 2010). However, in clinical practice, it is essential to be able to interpret findings at an individual level. The current study used a clinical measure of information processing to study WM fMRI activation in key regions of healthy controls, with a focus on individual level results. Specifically, we hypothesized that the SDMT would elicit activation in the corpus callosum and internal capsule in the majority of individuals.

## Methods

### Participants

Seventeen healthy adults provided written informed consent for their participation. The participants (9 F) had a mean age of 27.23 years (*SD* = 3.36). Fifteen participants were right-handed and two were left-handed. Individuals with contraindications for MRI were excluded, as were individuals on psychotropic medications or with neurological damage. We also set a priori exclusion criteria for individuals who demonstrated head motion that exceeded one voxel and for individuals who were unable to complete the task. The study was approved by the National Research Council board of ethics.

### Stimuli and procedure

The main objective in modifying standardized clinical tests for research purposes is to keep the adapted version as close to the clinical administration as possible (Connolly and D'arcy, 2000). During the clinical written SDMT, the patient is asked to use a legend to fill in numbers that match with symbol/number pairs in a legend with a 90 s limit (Smith, 1982). The SDMT had recently been adapted for use with fMRI (e.g., Genova et al., 2009; Kohl et al., 2009). As in previous studies, the modified SDMT presented a legend involving the same symbol/number combinations as used in the clinical version. During active blocks, participants were shown a symbol/number combination below the legend and asked to respond whether the stimulus was a “match” or “not a match” with the legend using a hand held response pad. During rest blocks, participants fixated on the center of the screen.

The task and instructions were presented visually through back-projection to a screen mounted inside the bore (and viewed through a mirror mounted on the head coil) using E-Prime (Psychology Software Tools, Inc). The task was administered one time for each subject, and consisted of five active blocks (36 s) and five rest blocks (18 s), yielding a time of approximately 5 min. All subjects performed the clinical paper-and-pencil SDMT and a short practice of the adapted task prior to imaging. The SDMT was administered 15–20 min into the imaging session and fatigue was not shown to be an issue on a self-report exit questionnaire administered immediately following the session.

### Data acquisition

Data were acquired from a 4 T Varian INOVA whole body MRI system. Gradients were provided by a body coil (Tesla Engineering Ltd.) operating at a maximum of 35.5 mT/m at 120 T/m/s, and driven by 950 V amplifiers (PCI). The RF coil used was a TEM head coil (Bioengineering Inc.). All images were obtained within one 60-min session.

Functional MRI was conducted using an asymmetric spin-echo (ASE) spiral sequence that collects three images per slice per volume (Brewer et al., 2009). The three ASE spiral images have equal blood-oxygen level-dependent (BOLD) contrast, but increasing T_2_-weighting. Prior work has shown that increased T_2_-weighting improves sensitivity to WM fMRI activation (Brewer et al., 2009; Gawryluk et al., 2009). Accordingly, the three ASE images were combined using an inverted signal weighted averaging algorithm. A total of 26 slices were acquired, which allowed for whole brain coverage with the following parameters: 5 mm axial slices, 0.5 mm gap, 64 × 64 matrix (220 × 220 mm), 1 shot, *TR* = 3 s, *TI* = 1400 ms, *TE* = 68 ms, and TE^*^ = 28 ms (where TE is the spin echo center and TE^*^ is the asymmetric echo time).

For structural registration purposes, a 3D T1-weighted FLASH whole brain anatomical image was collected. The parameters were as follows: *TR* = 10 ms, *TI* = 700 ms, *TE* = 5 ms, flip 11°, and 256 × 224 × 80 matrix (220 × 192 × 160 mm).

### Data analyses

Motion correction was carried out using SPM software (Friston et al., 1995; Oakes et al., 2005). Motion parameters were examined carefully for each subject to ensure that motion was not correlated with the task. Other pre-statistics processing steps were performed in FMRIB Software Library (FSL) using fMRI expert analysis tool (FEAT) version 5.3 (Smith et al., 2004; Woolrich et al., 2009). These steps included non-brain removal using BET (Smith, 2002), spatial smoothing using a Gaussian kernel of FWHM 5 mm (analyses were also performed without smoothing), mean-based intensity normalization of all volumes by the same factor, and highpass temporal filtering (100 s cutoff). Statistical analyses were performed using a model-based approach (General Linear Model). Time-series statistical analysis was carried out using FILM with local autocorrelation correction (Woolrich et al., 2001). Statistical thresholding was performed in FEAT using a cluster-based approach that is corrected for multiple comparisons. Z statistic images were reported using a corrected threshold for clusters determined by *Z* > 2.3 and a cluster significance threshold of *P* = 0.05 (Worsley et al., 1992). FLIRT was used to register functional data to anatomical images (DOF = 7) and to register anatomic images to the Montreal Neurological Institute template [12 DOF (Jenkinson and Smith, 2001; Jenkinson et al., 2002)]. Subsequently, in a similar approach to Mazerolle et al. (2013), FNIRT was used to refine the registration to standard space (Andersson et al., 2007a,b). Activation maps were displayed in FSLView (*Z* > 2.5).

Analyses were performed at the individual and group levels, although individual analyses were focused upon, in order to capture variability that is relevant to future patient studies/applications. To verify WM fMRI activation, individual data were examined against both the anatomic underlay and the raw spiral images (task vs. rest). The local maxima of clusters in the corpus callosum and internal capsule were also determined (using an increased threshold approach) to ensure that the cluster was centered in white matter. Subsequently, masks of the corpus callosum and internal capsule (based off of the JHU WM labels atlas) were tailored to each individual (i.e., the masks were examined for each subject and if areas outside of the regions of interest were captured, the masks were manually trimmed according to the subject's anatomy) and applied using pre-threshold masking to examine these regions of interest (ROIs).

In order to examine the relationship between groups with different levels of WM activation and behavioral data, split halves *t*-tests were performed using Statistical Package for the Social Sciences (SPSS).

## Results

### Functional MRI results

WM activation was present in 88% of participants (15/17). The activation was in either the corpus callosum (anterior and/or posterior) or internal capsule (left and/or right). Fifteen participants showed activation in the corpus callosum (7 anterior, 5 posterior, 3 both anterior, and posterior). Eight of these participants also showed activation in the internal capsule.

**Figure 1:**
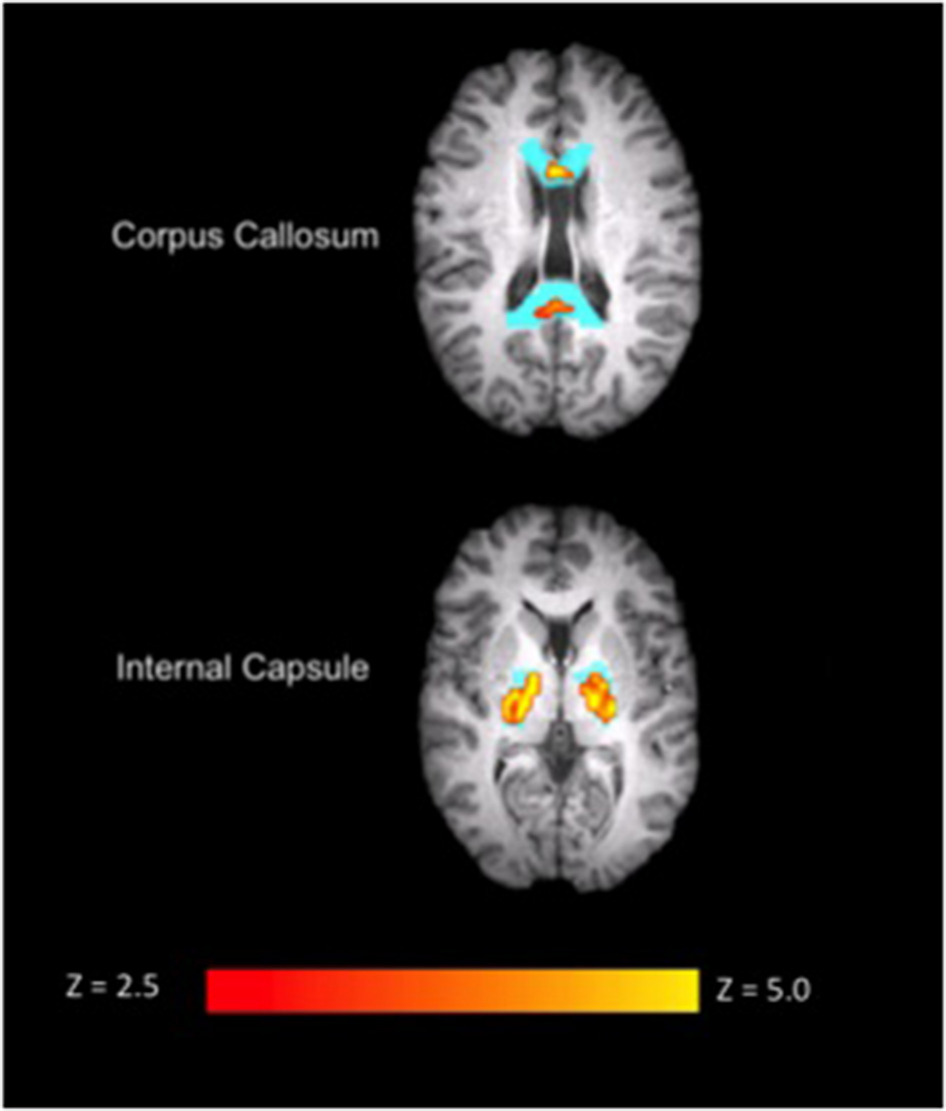
**Corpus callosum (top) and internal capsule (bottom) ROI results overlaid on anatomical data for a single subject (S9) during the SDMT**. The ROI mask is shown in blue. Images are in radiological view. Activation related to the task is displayed in red-yellow with a Z threshold of 2.5 to more clearly depict the activation.

Table 1 details the extent and maximum intensity of activation in the corpus callosum and internal capsule for each subject. Figure 1 shows the results of the ROI analyses for an illustrative subject overlaid on the subject's anatomical.

**Table 1:** **The extent and maximum intensity of activation in the corpus callosum (CC) and internal capsule (IC) using a cluster-based threshold (*z* > 2.3, *p* < 0.05), behavioral scores and demographic data for 15/17 subjects with white matter activation**.

**Subject**	**Number voxels cc**	**max z cc**	**Number voxels IC**	**max z IC**	**fMRI-SDMT ACC (%)**	**fMRI-SDMT RT(ms)**	**Wrritten SDMT**	**Handedness**	**Age (years, months)**	**Sex**
1	15	3.19	28	5.78	97	1635.30	83/83	Right	24 y,8 m	M
2	55	5.87	29	4.13	93	1119.22	58/58	Right	27 y, 5 m	M
3	14	4.60	21	3.06	97	1588.90	53/54	Right	29 y, 11 m	F
4	21	4.26	18	4.75	93	1447.65	49/51	Right	26 y, 9 m	M
5	47	4.51	158	5.31	97	1172.05	74/75	Right	30 y, 6 m	M
6	31	6.99	17	5.00	97	1108.13	76/78	Right	31 y, 1 m	F
7	126	8.04	12	4.34	87	1618.68	62/63	Right	20 y, 2 m	F
8	30	3.86	12	3.61	97	1541.50	56/60	Left	27 y, 9 m	M
9	17	3.85	None		93	1351.02	61/63	Right	25 y, 10 m	F
10	12	3.91	None		90	1365.45	72/72	Right	21 y, 7 m	F
11	29	4.52	None		77	1695.98	57/57	Right	32 y, 1 m	M
12	21	3.91	None		90	1321.63	91/93	Right	26 y, 8 m	M
13	7	3.74	None		93	1427.95	65/66	Right	31 y, 5 m	F
14	41	4.60	None		87	1502.47	59/59	Right	28 y, 2 m	M
15	50	5.78	None		83	1494.47	49/49	right	25 y, 10 m	F
Group	34.40	4.78	36.88	4.50	91.40	1426.03	64/65	14R, 1L	27 y, 6 m	7 F, 8 M

**Figure 2:**
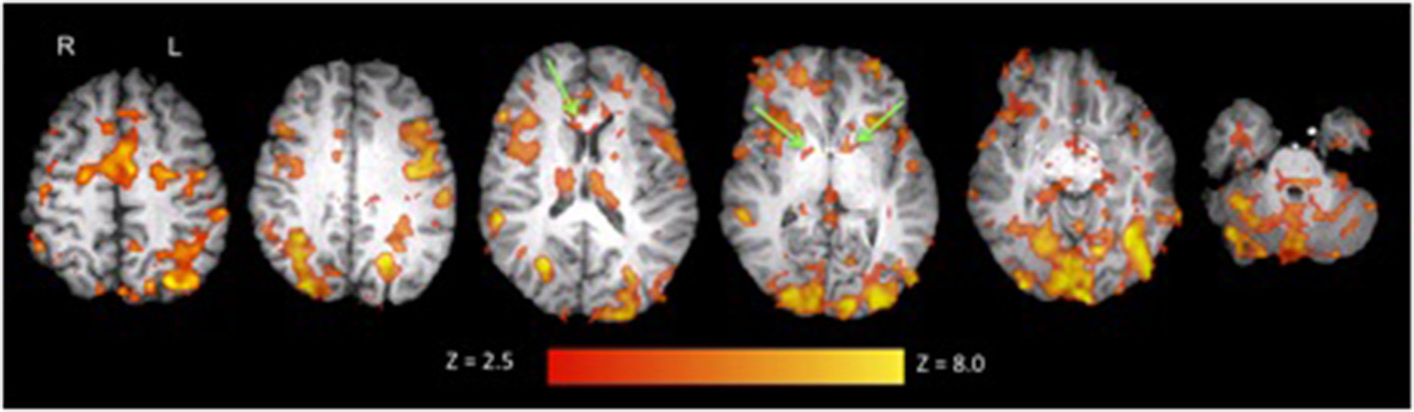
**Activation in white and gray matter during the adapted SDMT overlaid on anatomical data for a representative individual (S3)**. Activation clusters in the corpus callosum and internal capsule are pointed out in green. Images are in radiological view. Activation related to the task is displayed in red-yellow with a Z threshold of 2.5 to more clearly depict the activation.

Gray matter activation was observed at the individual level in occipital, parietal, temporal and frontal regions (including regions associated with visual stimulation and motor activation), as well as in the cerebellum. Figure 2 shows whole brain activation results for a representative individual (overlaid on the subject's anatomical).

**Figure 3:**
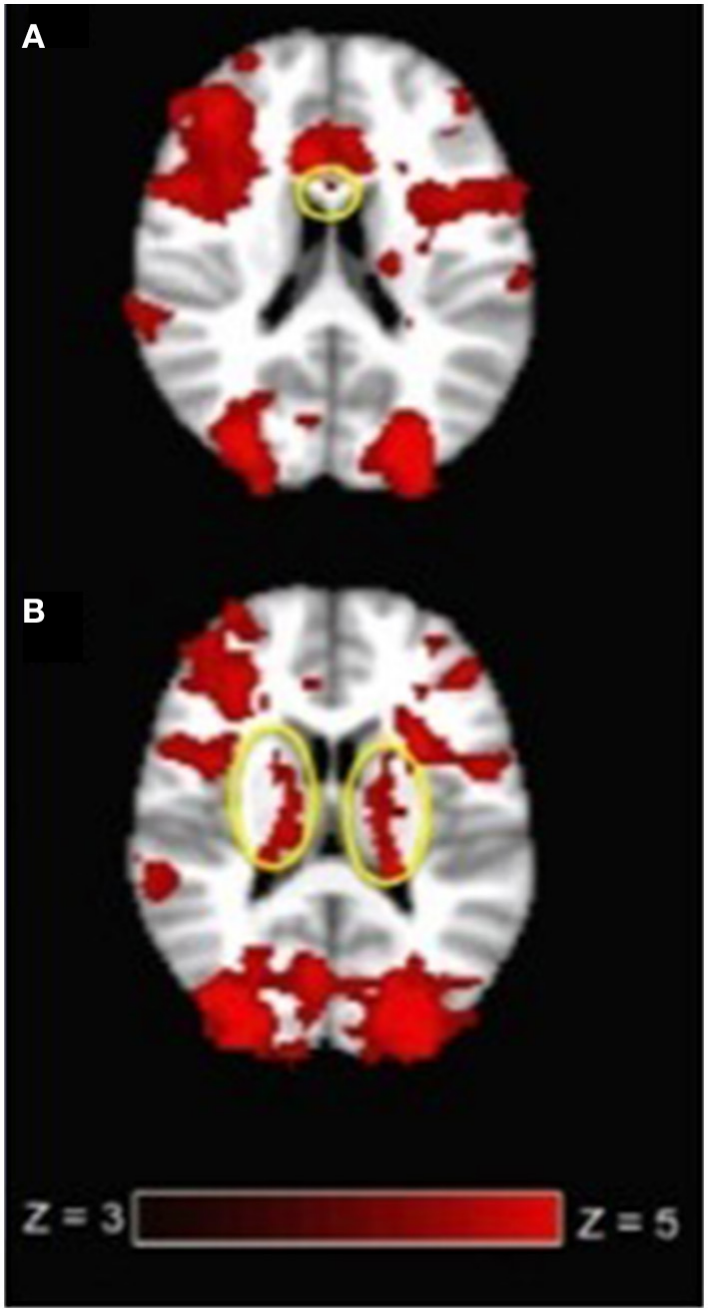
**(A)** Corpus callosum activation (circled in yellow) and **(B)** internal capsule activation (circled in yellow) at the group level (*N* = 17) during the SDMT. Images are in radiological view. Activation related to the task is displayed in red-yellow with a Z threshold of 3.0 to more clearly depict the activation.

The SDMT task revealed white matter activation at the group level in both the corpus callosum and internal capsule (Figure 3). The gray matter results at the group level mirror those depicted at the individual level (Figure 2).

### Behavioral results

Analyses of behavioral data demonstrated that participants performed the fMRI adapted SDMT with a mean accuracy of 91.71% (*SD* = 5.59%). The mean reaction time was 1432.00 ms (*SD* = 175.55 ms). The clinical written SDMT revealed that all participants' scores were in the normal range (mean = 62.88, *SD* = 12.33). A split halves *t*-test revealed a significant difference in fMRI accuracy scores between the group with activation in only the corpus callosum (mean = 87.57, *SD* = 5.83) and in both the corpus callosum and internal capsule (mean = 94.75, *SD* = 3.62, *p* = 0.01).

## Discussion

The current study evaluated whether the adapted SDMT elicited WM fMRI activation in healthy controls. As predicted, activation was observed in the corpus callosum and internal capsule for 88% of individuals and at the group level (*N* = 17).

These findings are consistent with previous fMRI results for the SDMT that were shown but not reported [36]. Importantly, the gray matter activation observed in the current study is also consistent with previous studies, which revealed activation in occipital, parietal, temporal, and frontal regions and the cerebellum (Forn et al., 2009; Genova et al., 2009).

The WM activation clusters were also generally consistent with the SDMT task requirements. For instance, the anterior corpus callosum is known to connect to pre-frontal and motor cortical areas, which fit with both the decision making component and motor response required (Stančák et al., 2000; Iacoboni and Zaidel, 2004; Zarei et al., 2006). The posterior corpus callosum is thought to link parietal areas involved in sensory integration related to the visual-perceptual task demands (Zarei et al., 2006; Mazerolle et al., 2008). Activation in the internal capsule, which contains corticospinal fibers and is thought to directly connect to the primary motor cortex (Nolte, 2002), which is also consistent with the motor component of the task. Importantly, variability between subjects has previously been demonstrated in gray matter (Miller et al., 2009). Such variability might be based on differences in underlying vasculature, regional/individual differences in hemodynamic response functions, or differences in strategy or effort during the task. This type of variability may be difficult to detect when testing is limited to traditional behaviorally-based measures and will be a key consideration in future patient studies/applications. In order to examine the relationship between behavioral scores and fMRI results, the individuals with activation in the CC and IC and those with activation in the CC alone were compared. The results revealed lower accuracy scores in the group with less white matter activation, which could potentially reflect the strategy or level of effort put forth/required during the adapted SDMT task.

Interpretation of white matter fMRI activation might be further improved by evaluating its relationship to gray matter. In a previous study, we used an interhemispheric transfer task that elicited activation in both hemispheres as well as the corpus callosum. We also collected DTI data and showed that tracts connecting the activation in each hemisphere were co-localized to the white matter activation (Mazerolle et al., 2010). Thus, there is an anatomical substrate by which white and gray matter activation may be related. However, it will be important for future studies on WM fMRI to collect DTI data as well in order to allow for examination of the structural connections between different areas of activation (e.g., internal capsule). Furthermore, DTI data would allow for an examination of the relation between microstructural properties (e.g., fractional anisotropy) and white matter activation.

The functional connectivity among regions of white and gray matter activation area is also of interest. Recent work evaluated resting state functional connectivity within white matter tracts, showing temporal intervoxel correlations that demonstrated anisotropy (i.e., voxels were more correlated within a tract than with random voxels matched for distance; Ding et al., 2013). This approach could potentially be extended to study the functional connections between white and gray matter at rest. Understanding the relations between task-based white and gray matter activation is also an interesting avenue for future research. For example, whether the gray matter activation is predictive of signal changes in white matter could be explored.

Although WM represents approximately 50% of the tissue in the brain (Black, 2007), fMRI has rarely been investigated in this tissue. As mentioned, the idea of WM fMRI activation is controversial and such results are often ignored (Logothetis and Wandell, 2004). While we have shown that WM fMRI activation can be detected, characterized, and linked to a neuropsychological test, the current techniques may benefit from optimization.

In particular, it remains possible that data acquisition methods can be optimized for detection of WM fMRI activation. For example, we employed an ASE spiral sequence [which can provide increased sensitivity to WM activation; (Gawryluk et al., 2009)] and used 4T MRI, which is more sensitive than 1.5T MRI (Mazerolle et al., 2013). However, there are other studies that have used standard imaging sequences and reported white matter activation at 1.5T (e.g., Fabri et al., 2011). It may also be possible to increase sensitivity to white matter activation by using a white matter specific hemodynamic response function. Although there is some evidence that the hemodynamic response function in the corpus callosum resembles the canonical hemodynamic response function (Fraser et al., 2012), other work has demonstrated a slower response function in white matter (Yarkoni et al., 2009). Part of the difficulty in interpreting how and when white matter activation is detected is that some groups report these findings and others do not (e.g., Genova et al., 2009). This variability makes it difficult to assess when and with what types of parameters investigators are detecting white matter activation.

### Caveats

One limitation of the current study relates to the investigation of the relationship between the clinical and adapted SDMT. There are differences that exist between the tasks (e.g., with the legend replaced on each trial, the working memory component has been removed from the adapted version). However, it remains difficult to compare the two versions of the task given that the scoring of the tasks is inherently different. The current study used an adapted version of the SDMT that has been used in the literature (to confirm an unreported finding). Given the potential for this task to be of clinical use, future efforts may focus on validation or standardization.

Given that the detection of fMRI activation in white matter remains controversial, there is skepticism and concern regarding the nature of such findings. From an analysis perspective, registration can be particularly challenging when the focus is on examining small white matter and subcortical structures, such as the internal capsule. The current study used a non-linear approach to refine registration to standard space. Non-linear registration techniques have been used in diffusion tensor imaging studies, which also focus on white matter (Smith et al., 2007) and have been shown to improve subcortical registration (Chakravarty et al., 2009; Klein et al., 2009). Nevertheless, registration is a common concern, and there is still potential for misregistration caused by susceptibility-induced distortions in the functional images. Registration for a representative subject from the current study is shown in Supplemental Figure 1.

From a physiological perspective, a caveat of the current study relates to partial volume effects, which cannot be completely ruled out at the current spatial resolution. Future studies should use higher resolution data acquisition to minimize these effects. In terms of data analyses, the current data were also examined without smoothing applied with no changes to the results.

## Conclusions

The current study is the first to investigate WM fMRI activation associated with a clinical measure. The SDMT was implemented because it is commonly used in clinical settings to detect WM dysfunction (Hoffmann et al., 2007). Given that the fMRI adapted SDMT demonstrated WM activation in predicted regions, it shows potential as a clinical assessment tool. Given the individual variability in activation with this task, we speculate that the SDMT fMRI task may be best suited to tracking progression/changes within individuals in WM function over the course of diseases (i.e., longitudinal evaluations of patients). This idea is supported by previous studies that have demonstrated that the SDMT can be used to predict “clinically meaningful cognitive decline” (Morrow et al., 2010) and that it is the “most sensitive” test to measure cognitive decline longitudinally in patients with multiple sclerosis (Amato et al., 2010). The next step in this line of research is to use the fMRI adapted SDMT to test patients with WM disorder to further explore the clinical value of this technique.

## Author contributions

Jodie R. Gawryluk was involved in the conceptualization and design of the study, acquisition of data, analysis and interpretation of data and drafting the manuscript. Erin L. Mazerolle was involved in the acquisition of data, analysis and interpretation of data and revising the manuscript. Steven D. Beyea and Ryan C. N. D'Arcy were involved in the acquisition of data and critically revised the manuscript for intellectual content. All authors read and approved the final manuscript.

### Conflict of interest statement

The authors declare that the research was conducted in the absence of any commercial or financial relationships that could be construed as a potential conflict of interest.

## Supplementary material

Supplemental Figure 1 **An example of the registration from functional to standard space (as depicted by FSL) for a representative subject**.
