# Supplementary figures and images for: Functional MRI activation in white matter during the Symbol Digit Modalities Test
Source: Front Hum Neurosci. 2014 Aug 4;8:589. doi: 10.3389/fnhum.2014.00589 (PMC4120763; doi:10.3389/fnhum.2014.00589)

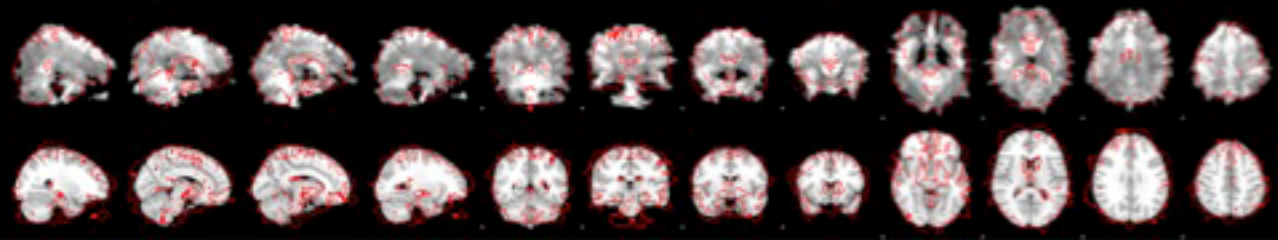

Supplement: Supplemental Figure 1 — An example of the registration from functional to standard space (as depicted by FSL) for a representative subject. [file Presentation1.PDF]
